# Involvement of *Anopheles nili* in *Plasmodium falciparum* transmission in North Benin

**DOI:** 10.1186/s12936-019-2792-0

**Published:** 2019-04-29

**Authors:** Razaki A. Ossè, Filémon Tokponnon, Germain Gil Padonou, Aboubakar Sidick, Rock Aïkpon, Arsène Fassinou, Come Z. Koukpo, Wilfrid Sèwadé, Bruno Akinro, Arthur Sovi, Melchior Aïssi, Martin C. Akogbéto

**Affiliations:** 1 Ecole de Gestion et d’Exploitation des Systèmes d’Elevage, Université Nationale d’Agriculture, Kétou, Benin; 2 Centre de Recherche Entomologique de Cotonou (CREC), 06 BP 2604 Cotonou, Benin; 3 National Malaria Control Programme, Ministry of Health, Cotonou, Benin; 4 Faculté des Sciences et Techniques, Université d’Abomey-Calavi, Abomey-Calavi, Benin; 5 Ecole Normale Supérieure de Natitingou, Université Nationale des Sciences, Technologies, Ingénierie et Mathématiques, Natitingou, Benin; 6 Faculté d’Agronomie, Université de Parakou, Parakou, Benin; 7 Conseil National de Lutte contre le SIDA, la Tuberculose, le Paludisme, les Hépatites et les épidémies, Cotonou, Benin

**Keywords:** Malaria, EIR, *Anopheles nili*, Contribution, Benin

## Abstract

**Background:**

Several studies carried out in Benin have shown the involvement of *Anopheles gambiae* sensu lato (s.l.), the *Anopheles funestus* group in malaria transmission, but none of them reported the contribution of the *Anopheles nili* group to the transmission of this disease. The current study investigated the question through an entomological cross-sectional survey performed in Northern Benin.

**Methods:**

Mosquito samplings were performed in September and October 2017 in 4 villages located in two districts: Bambaba and Wodara (Kérou district) and, Péhunco 2 and Béké (Péhunco district). The collections were carried out indoors and outdoors using human landing catches (HLC) to assess the human biting rate (HBR) and pyrethrum spray catches (PSC) to evaluate the blood feeding rate and the blood meal origin using the ELISA test. All collected mosquitoes were morphologically identified and, the polymerase chain reaction (PCR) technique was used for molecular identification of sibling species of *An. gambiae* s.l., *An. funestus* group and *An. nili* group sporozoite index (SI) was also assessed by the ELISA test.

**Results:**

Overall, *An. gambiae* s.l., *An. funestus* group and *An. nili* group were the three vectors found in the study area. A significantly higher human biting rate (HBR) was recorded in *An. nili* group (5 bites/human/night) compared to *An. funestus* group (0.656 bites/human/night) in the Kérou district (p < 0.0001). *Anopheles gambiae* s.l. displayed the highest HBR (26.19 bites/human/night) in the same district. The entomological inoculation rate (EIR) was 1.875 infected bites/human/month in *An. nili* group against 13.05 infected bites/human/month in *An. gambiae* s.l. and 0.938 infected bites/human/month in *An. funestus* group in Kérou. In Péhunco, the EIR was 1.02 infected bites/human/month in *An. gambiae* s.l. PCR results showed that *An. nili* sensu stricto (s.s.) and *An. funestus* s.s. were the only species of the *An. nili* and *An. funestus* groups, respectively. The anthropophagic character of *An. gambiae* s.l. was also highlighted.

**Conclusion:**

This study provides useful information on the contribution of *An. nili* group as secondary vector to malaria transmission in northern Benin. Broader studies must also be carried out in a larger study area to assess the involvement of other *Anopheles* species to malaria transmission. This will aid to better plan malaria vector control interventions.

## Background

A wide range of human and animal diseases are transmitted by mosquitoes and those diseases remain a major source of death worldwide [1]. Malaria remains one of the most serious vector-borne diseases, affecting half of the world’s 7.4 billion people [2]. The proliferation of mosquitoes is promoted not only by ecological changes due to human activities (deforestation, public works, construction of dams, rice paddies, irrigation), but also by environmental parameters (rainfall, temperature and relative humidity), which also play a fundamental role in the level of transmission and the epidemiology of diseases [3, 4]. In intertropical Africa, malaria transmission is very heterogeneous due to eco-climatic variations [5]. Currently, five species of the parasite of the *Plasmodium* genus have been identified as responsible for malaria infection in humans [6]. Among them *Plasmodium falciparum* remains the most virulent species causing the deadly forms of malaria [7]. The *Plasmodium* species responsible for human malaria are mainly transmitted by primary vector species, such as *Anopheles gambiae* sensu lato (s.l.), *Anopheles funestus* group and *Anopheles nili* group [8, 9]. The existence of those different species complexes in an area represents a great challenge for malaria control programmes. *Anopheles nili* group has a wide geographical distribution in most of tropical Africa [10] and its preferential habitats are fast-flowing, upright-flowing streams, large rivers or dense shade along streams [7]. It is a group that includes four species, including *An. nili* sensu stricto (s.s.), *Anopheles carnevalei*, *Anopheles somalicus* and *Anopheles ovengensis* [8, 11].

In Benin, over the past two decades, the roles of *An. gambiae* s.l. and *An. funestus* group in the transmission of *P. falciparum* in several regions of the country have been studied by several authors [12–19]. Few of these studies have shown the presence of *An. nili* group in the different surveyed areas. Moreover, none of them has demonstrated its involvement in malaria transmission in Benin. It is in this context that a large population of *An. nili* group was collected during this cross-sectional survey conducted in the districts of Kérou and Péhunco located in the Atacora region. Thus, the possible involvement of this *Anopheles* species in the transmission of *P. falciparum* was investigated in the study area.

This study aims at providing information on vector species composition involved in malaria transmission in the areas of Kérou and Péhunco (northwestern Benin) where there is a lack of data.

## Methods

### Study area

**Fig. 1:**
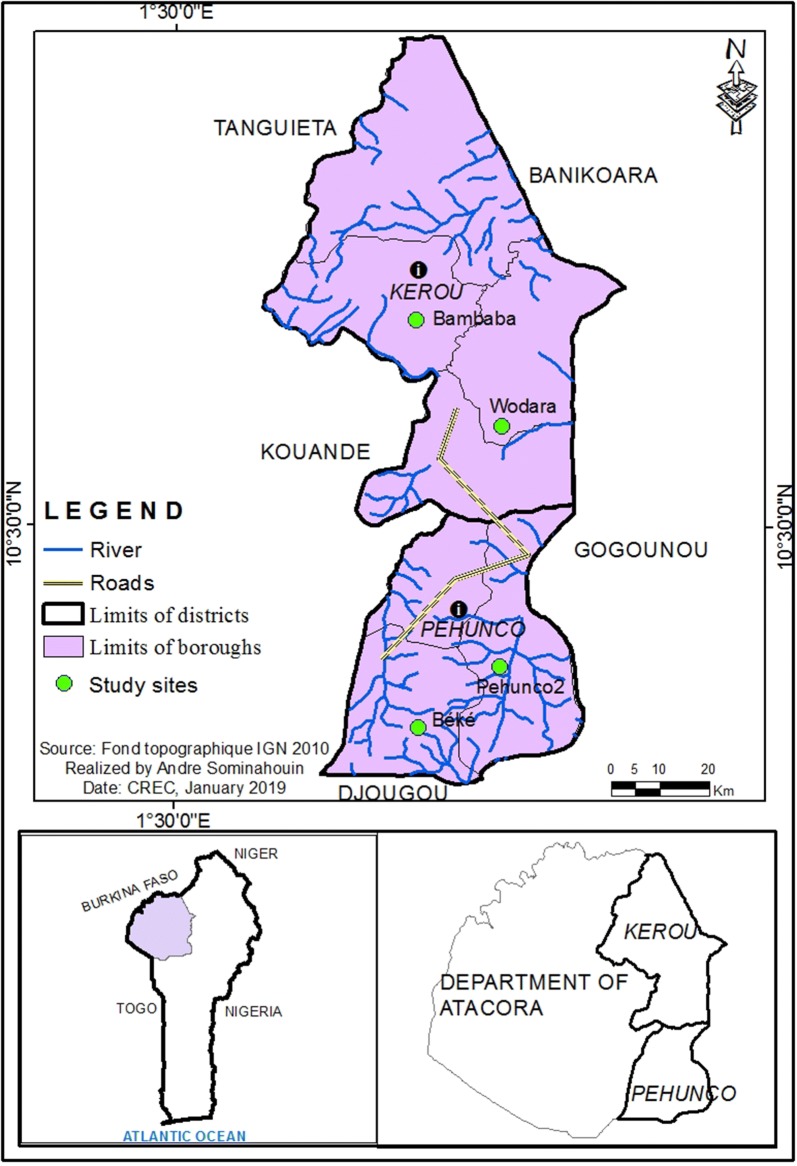
Map of the study area

This study was conducted from September to October 2017 in the districts of Kérou and Péhunco, both located in the north-western Benin (Fig. 1). The two districts have a Sudano-Guinean climate characterized by a rainy season, from mid-April to mid-October, and a dry season from mid-October to mid-April. They belong to an agroecological zone characterized by an average annual rainfall of 1000 mm. The average temperature varies between 25 in August and 31 °C in April.

The district of Kérou is irrigated by two large permanent rivers, the Mekrou and the Pendjari as well as of many tributaries from the Alibori river. Péhunco is drained by the river Mekrou and also by many rivers. The most important economic activity of these two districts is agriculture, especially with the cultivation of cotton, sorghum and cashew nuts [20].

### Mosquito sampling and processing on the field

In each district, two villages were selected for mosquito collections, Wodara and Bambaba (respectively central and peripheral village of the Kérou district) and, Péhunco 2 and Béké (respectively central and peripheral village of the Péhunco district). Collections of mosquitoes were performed using both human landing catches (HLC) and pyrethrum spray catches (PSC).

HLCs were carried out in two houses per village. Four sessions of night collections were organized each month with one human sitting inside and another one outside of each house, bringing to a total of 16 human-nights/village/month and a total of 32 human-nights/district/month.

For PSCs, 10 bedrooms were surveyed in each district (5 in the central village and the remaining 5 in the peripheral village) to collect early in the mornings, all mosquitoes that have entered the houses the night before. Thus, aerosol bombs (Rambo^®^) containing 0.25% transfluthrin and 0.20% permethrin were sprayed in the houses and white canvas were spread on the floor for the collection of fallen mosquitoes.

Mosquitoes caught by both methods were morphologically identified using a mosquito identification key [21] and, the ovaries of the *Anopheles* vectors were dissected to determine the parity rate [22]. *Anopheles* vectors collected by PSCs were classified according to the physiological state of their abdomens (unfed, fed, half gravid, gravid) to determine the blood feeding rate. The *Anopheles* vectors collected with the two sampling methods were then stored in tagged eppendorf tubes containing silica gel and cotton for subsequent laboratory analysis.

### Laboratory analysis

539 head-thoraxes of *Anopheles* vectors collected indoor and outdoor through HLC were crushed and then analysed by the ELISA tests for the determination of the circumsporozoite protein (CSP) positivity of *Plasmodium falciparum* using the protocol of Wirtz et al. [23]. This allows the determination of the sporozoite index (SI). The legs, wings, and abdomens of these mosquitoes were used for DNA extraction to perform molecular species identification.

The PCR technique based on the protocols of Santomalazza et al. [24], Koekemoer et al. [25] and Kengne et al. [26] was used to identify the sibling species of *An. gambiae* complex, *An. funestus* group and *An. nili* group, respectively. In *An. gambiae* s.l., the presence of L1014F *kdr* and G119S *ace*-*1* mutations was determined following the methods of Martinez-Torres et al. [27] and Weill et al. [28], respectively. Moreover, the blood meal origin was sought in the blood fed *Anopheles* mosquitoes collected by PSC, using a direct enzyme immunoassay (ELISA) according to the method of Beier et al. [29] with human, cattle, sheep, chicken and pig antibodies.

### Data analysis

The results were processed and analysed using the R Core Team software (Version 3.5.1-2018) and Excel spreadsheets. The human biting rates (HBR = number of collected vectors/number of humans/number of nights), infection rates (Number of infected mosquitoes/total tested), entomological inoculation rates (EIR_night_ = HBR × infection rate; EIR_month_ = EIR_night_ × 30) and parity rates (Number of parous mosquitoes/total tested) were calculated and compared between species of the same district. The Poisson test allowed us to compare the HBRs and EIRs between species in the district of Kérou. The comparison of parity rates, infectivity and allelic frequencies of L1014F *kdr* and G119S *ace*-*1* mutations by species and by site were made with the Chi square comparison test.

## Results

### Mosquito species composition and molecular identification of sibling species of *Anopheles gambiae* complex, *Anopheles funestus* group and *Anopheles nili* group

The mosquito fauna collected in both surveyed districts was very diverse (Table 1). A total of 1558 mosquitoes divided into 12 different species were collected with a predominance of *Anopheles* species (83.25%: 1297/1558).

**Table 1:** Mosquito species composition in Péhunco and Kérou between September and October 2017

Species	Péhunco		Kérou		Total
	Indoor	Outdoor	Indoor	Outdoor	
*Anopheles gambiae* s.l.	74	54	430	408	966
*Anopheles funestus* group	0	1	9	12	22
*Anopheles nili* group	0	0	51	109	160
*Anopheles pharoensis*	0	0	0	1	1
*Anopheles ziemanni*	0	0	14	33	47
*Anopheles brohieri*	0	0	19	82	101
*Anophelinae* (sub total 1)	74	55	523	645	1297
*Aedes aegypti*	0	0	1	0	1
*Aedes vitatus*	0	0	0	2	2
*Culex quinquefasciatus*	127	91	18	16	252
*Culex nebulosus*	0	2	0	0	2
*Culex tigripes*	1	0	0	0	1
*Mansonia africana*	1	1	0	1	3
*Culicinae* (sub total 2)	129	94	19	19	261
Grand total	203	149	542	664	1558

In Péhunco, out of the collected mosquitoes, 36.36% (128/352) were *An. gambiae* s.l. and 0.3% (1/352) *An. funestus* group. In the Kérou district, 9 species were collected. Among those species, *An. gambiae* s.l. was the most abundant (69.48%: 838/1206) followed by *An. nili* group (13.27%: 160/1206), *Anopheles brohieri* (8.37%: 101/1206), *Anopheles ziemanni* (3.90%: 47/1206) and *An. funestus* group (1.74%: 21/1206).

**Fig. 2:**
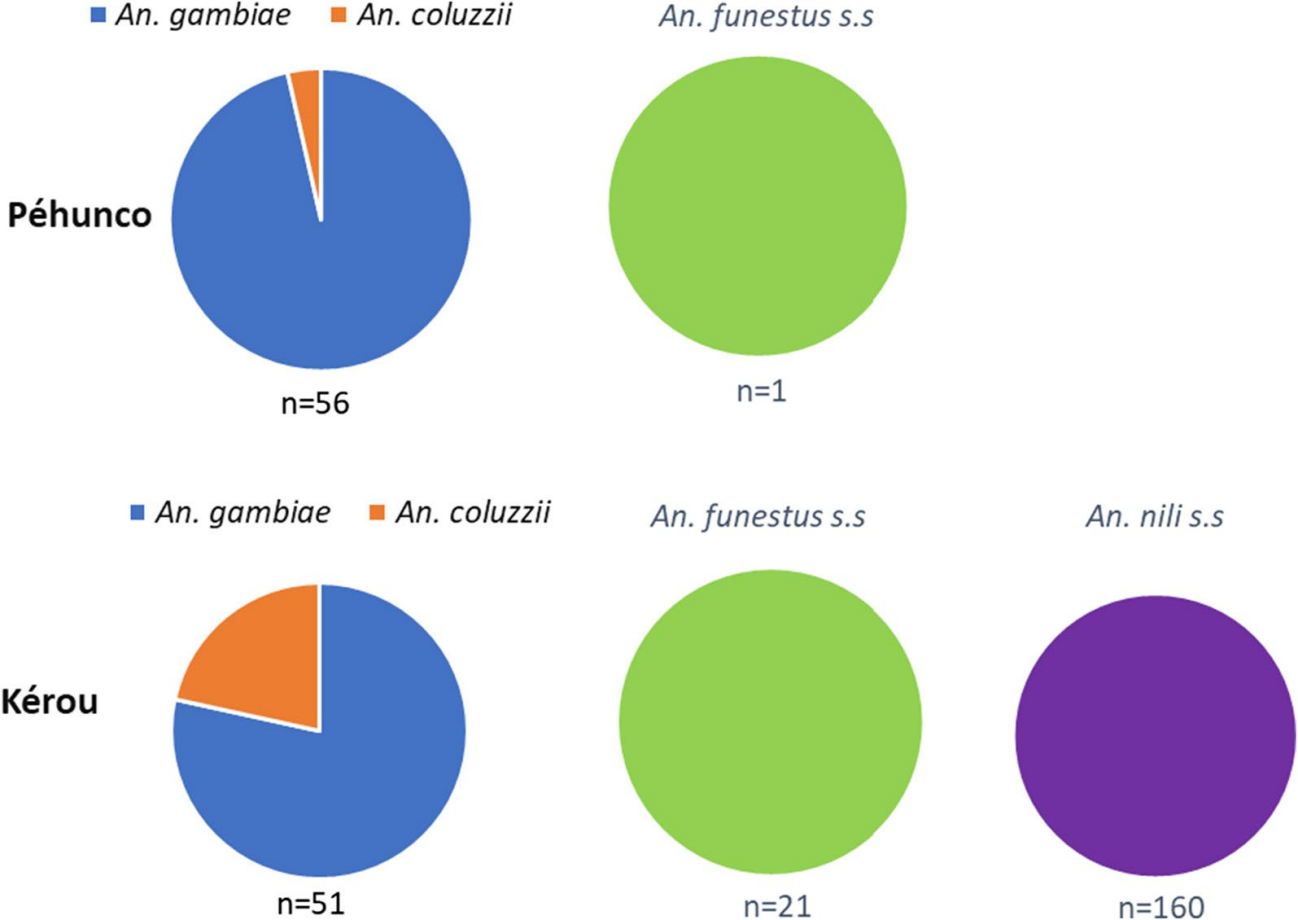
Distribution of sibling species within the *An. gambiae* complex and the *An. funestus* and *An. nili* groups in the study sites

PCR results showed the presence of *An. gambiae* and *An. coluzzii* as sibling species of the *An. gambiae* complex in both Péhunco and Kérou. *Anopheles gambiae* was found in majority (96.43% and 78.43% at Péhunco and Kérou, respectively). In addition, all mosquito specimens of the *An. funestus* group (Péhunco and Kérou) and the *An. nili* group (Kérou) were found to be *An. funestus* s.s. and *An. nili* s.s., respectively (Fig. 2).

### Biting behaviour, HBR, SI and EIR

In Kérou, the *An. nili* group was exophagic as the proportion of this species caught outdoors (68.13%: 109/160) was significantly higher than that recorded indoors (31.87%: 51/160) (p < 0.0001). In the same district the indoor (51.31%: 430/838) and the outdoor (48.69%: 408/838) biting behaviour of *An. gambiae* s.l. was similar (p = 0.282), as was also observed for the *An. funestus* group [indoors proportion: 42.86% (9/21); outdoors proportion: 57.14% (12/21); p = 0.35]. However, in Pehunco, *An. gambiae* s.l. was endophagic [Indoors proportion: 57.81% (74/128); Outdoors proportion: 42.19% (54/128); p = 0.011] (Table 1).

In the district of Kérou, the biting rate of *An. gambiae* s.l. (26 bites/human/night) was significantly higher than that of the *An. nili* group (5 bites/human/night) (p < 0.0001). The biting rate of *An. funestus* group (0.656 bites/human/night) was significantly lower than that of *An. gambiae* s.l. and *An. nili* group (p < 0.0001). The trend was the same between *An. funestus* group and *An. gambiae* s.l. in Péhunco (p < 0.0001) (Table 2).

**Table 2:** Entomological inoculation rate (EIR) in *Anopheles gambiae* s.l., *Anopheles funestus* group and *Anopheles nili* group in the Péhunco and Kérou districts

	Biting locations	Number of collected mosquitoes		HBR (b/h/night)		Sporozoite index (N tested)		EIR (ib/h/night)		EIR (ib/h/month)	
		Kérou	Péhunco	Kérou	Péhunco	Kérou	Péhunco	Kérou	Péhunco	Kérou	Péhunco
*An. gambiae* s.l.	Indoors	430	74	26.875^a^	4.625^a^	0.025 (121)^a^	0.014 (66)^a^	0.672	0.065	20.156^a^	1.943^a^
	Outdoors	408	54	25.5^a^	3.375^a^	0.008 (120)^a^	0 (50)^a^	0.204	0	6.120^b^	0^b^
	Total	838	128	26.19*	4*	0.017* (241)	0.009 (116)	0.435	0.034	13.05*	1.02
*An. funestus* group	Indoors	9	0	0.5625^b^	0^a^	0.111 (9)^a^	0	0.062	0	1.873^c^	0
	Outdoors	12	1	0.75^b^	0.0625^a^	0 (12)^a^	0 (1)	0	0	0^d^	0
	Total	21	1	0.656^‡^	0.031^‡^	0.048 (21)*	0 (1)	0.031	0	0.9375^‡^	0
*An. nili* group	Indoors	51	0	3.1875^c^	0	0.02 (51)^a^	0	0.064	0	1.913^e^	0
	Outdoors	109	0	6.8125^d^	0	0.009 (109)^a^	0	0.061	0	1.839^e^	0
	Total	160	0	5^¥^	0	0.0125 (160)*	0	0.063	0	1.875^¥^	0

b/h, bite/human; ib/h, infected bite/human; N, number of mosquitoes

^a,b,c,d,e^Values with different superscripts at the two biting locations of the same district are significantly different (p < 0.05)

^*,‡,¥^The total values with different superscripts in a same district are significantly different (p < 0.05)

For the SI, no significant difference was recorded between the three vectors in the Kérou district (p = 0.496). In Péhunco, only *An. gambiae* s.l. was found positive for *P. falciparum* antigen (Table 2).

In Kérou, the indoors EIR of *An. gambiae* s.l. (20.156 infected bites/human/month) and *An. funestus* group (1.873 infected bites/human/month) were significantly higher than that recorded outdoors [*An. gambiae* s.l.: 6.12 infected bites/human/month (p < 0.0001); *An. funestus* group: 0 infected bites/human/month (p = 0.0005)]. In the same district, the indoor (1.913 infected bites/human/month) and the outdoor (1.839 infected bites/human/month) EIR of *An. nili* group was similar (p = 1). Furthermore, in the district of Kérou, the malaria transmission risk by *An. gambiae* s.l. (13.05 infected bites/human/month) was significantly higher than the malaria transmission risk of the *An. nili* group (1.875 infectious bites/human/month) (p = 0.0009) and the *An. funestus* group (0.938 infected bites/human/month) (p < 0.0001). It should be noted that the EIR of *An. nili* group was significantly higher than that of *An. funestus* group in Kérou (p < 0.0001) during the study period. In Péhunco, the indoor EIR for *An. gambiae* s.l. (1.943 infected bites/human/month) was significantly higher than that noted outdoor (0 infected bites per month) (p < 0.0001) (Table 2).

### Parity rate of *An. gambiae* s.l., *An. funestus* group and *An. nili* group

**Fig. 3:**
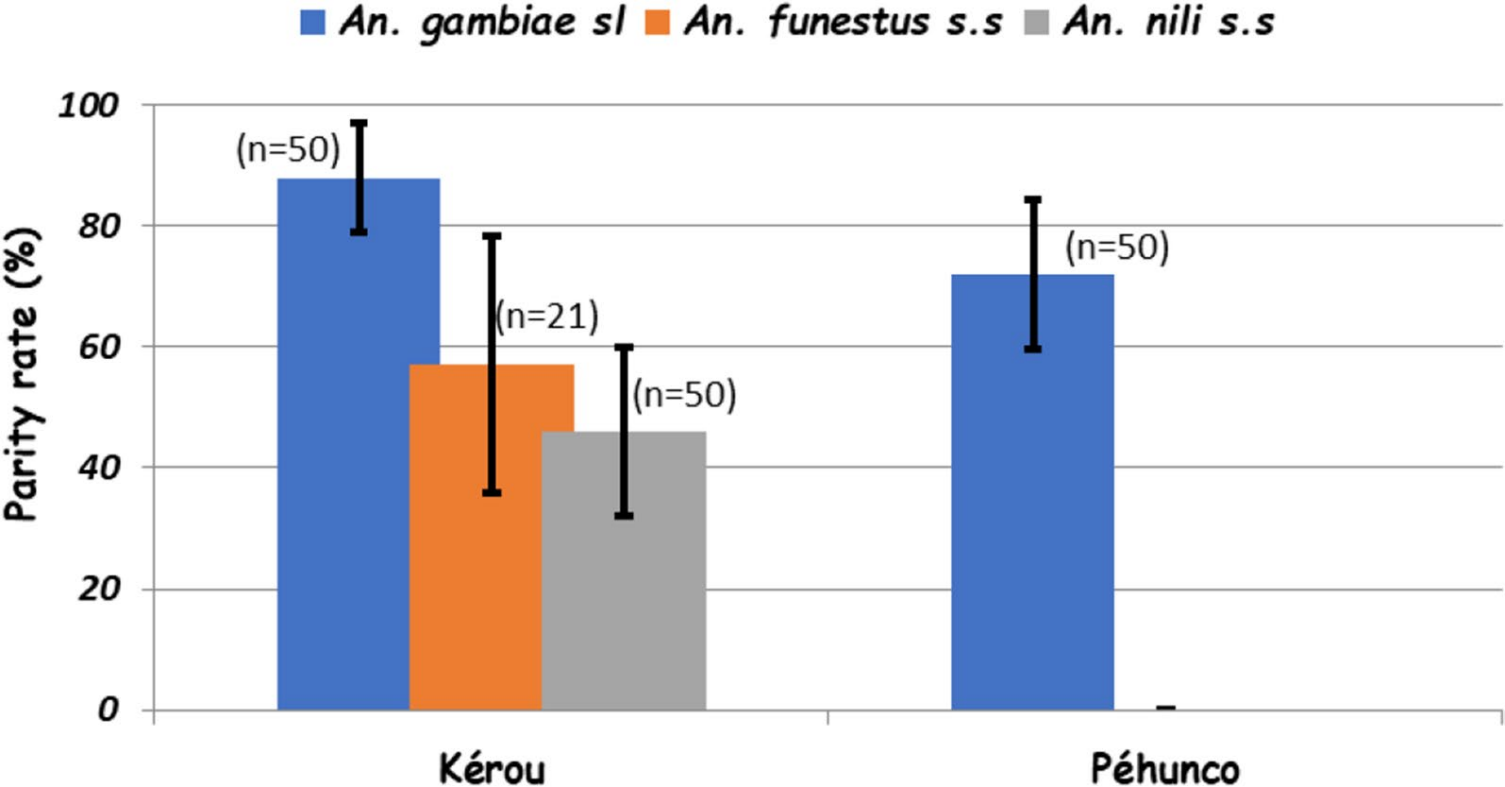
Parity rate in *An. gambiae* s.l., *An. funestus* s.s. and *An. nili* s.s. collected in Kérou and Péhunco

Figure 3 shows the different parity rates with 88% [78.99–97.01], 57.14% [35.97–78.31] and 46% [32.19–59.81] for *An. gambiae* s.l., *An. funestus* group and *An. nili* group in the Kérou district, respectively. The parity rate in *An. gambiae* s.l. was significantly higher than that of *An. funestus* group (p = 0.004) and *An. nili* group (p < 0.0001). In Pehunco, the parity rate recorded for *An. gambiae* s.l. was 72% [59.55–84.45].

### Blood feeding rate and blood meal source in *An. gambiae* s.l

The blood feeding rate of *An. gambiae* s.l. collected inside houses after PSC was 63.16% in Péhunco against 60.53% in Kérou (p = 0.847). In both districts, the majority of *An. gambiae* s.l. vectors took their blood meal on human (91.30% in Kérou against 100% in Péhunco). In Kérou, 4.35% (1/23) of the collected *An. gambiae* s.l. blood fed on beef and, the same proportion on sheep (Table 3).

**Table 3:** Blood meal origin in *Anopheles gambiae* s.l. collected indoors in Kérou and Péhunco

Districts	T. collected	T. Blood fed	Blood feeding rate (%)	Blood meal origin in *An. gambiae* s.l.			
				N. Human (%)	N. Beef (%)	N. Sheep (%)	N. Pig (%)
Péhunco	19	12	63.16	12 (100)			0
Kérou	38	23	60.53	21 (91.30)	1 (4.35)	1 (4.35)	0
Total	57	35	61.40	22 (62.86)	1 (2.86)	1 (2.86)	0

T, total of mosquitoes; N., number of mosquitoes having taken their blood meal on

### L1014F *kdr* and G119S *ace*-*1* allele frequencies in *An. gambiae* and *An. coluzzii*

The L1014F *kdr* mutation was found at very high frequencies in both Péhunco (86% and 100% respectively in *An. gambiae* and *An. coluzzii)* and Kérou (83% and 73% respectively in *An. gambiae* and *An. coluzzii)* (Table 4). In each district, the allelic frequencies of the L1014F *kdr* mutation were similar in *An. gambiae* and *An. coluzzii* (p > 0.05).

**Table 4:** Allelic frequencies of L1014F *kdr* and G119S *ace*-*1* mutations in *Anopheles gambiae* and *Anopheles coluzzii* in the Péhunco and Kérou districts

	Species	N tested	L1014F *Kdr*				*p* value	G119S *Ace*-*1*				*p*-value
			RR	RS	SS	F(L1014F *Kdr*)		RR	RS	SS	F(G119S *Ace*-*1*)	
Péhunco	*An. gambiae*	54	42	9	3	0.86	0.957	0	5	49	0.05	1
	*An. coluzzii*	2	2	0	0	1		0	0	2	0	
Kérou	*An. gambiae*	41	30	8	3	0.83	0.439	0	6	35	0.073	1
	*An. coluzzii*	11	7	2	2	0.73		0	1	10	0.05	

The G119S *ace*-*1* mutation was also recorded in the two districts but at low levels with allelic frequencies of 5% and 0%, respectively, in *An. gambiae* and *An. coluzzii* in Péhunco and, 7.3% and 5%, respectively, in *An. gambiae* and *An. coluzzii* in Kérou (Table 4). No significant difference was also recorded between the allelic frequencies of the G119S *ace*-*1* mutation of *An. coluzzii* and *An. gambiae* in both districts (p > 0.05).

## Discussion

The study of malaria vectors in an area is a prerequisite not only to understand the epidemiology of the disease but also to implement a strategy for effective and targeted control of those vectors. In the current study, 12 mosquito species including 6 anophelinae were identified in the two surveyed districts. Among these 6 *Anopheles* species, two (*An. gambiae* s.l. and *An. funestus* group) have already been implicated in malaria transmission in Benin [13, 17, 18]. During this cross-sectional study, they were found in both study districts but, *An. nili* group was found only in Kérou. The three vector species (*An. gambiae* s.l., *An. funestus* group and *An. nili* group) identified in Kérou live in sympatry in this savannah zone. This finding is similar to those of several authors in West Africa [30, 31].

In Péhunco and Kérou, *An. gambiae* and *An. coluzzii* are the only members of the *An. gambiae* complex recorded with a predominance of *An. gambiae* (96.43% and 78.43%, respectively, in Péhunco and Kérou). No species of *An. arabiensis* was identified after PCR in both districts. This could be due to anthropogenic actions such as deforestation and urbanization that destroy its natural habitats causing its gradual disappearance in some areas of northern Benin as reported by Aïkpon et al. [32] and Salako et al. [33]. All the specimens of the *An. nili* and *An. funestus* groups identified by PCR were *An. nili* s.s. and *An. funestus* s.s. in the district of Kérou. These results corroborate those of Djouaka et al. [19, 34] who found *An. funestus* s.s. as the only species of the *An. funestus* group in the areas of Pahou and Kpomè in southern Benin. The results are also similar to works by Carnevale et al. [35], Dia et al. [36] and Adja et al. [31], who found *An. nili* s.s. as the only species in forest zone respectively in Cameroon, Senegal and Côte d’Ivoire.

The number of *An. funestus* group collected in both districts was low during the study period as was also reported by some authors in the area and around [32, 37, 38]. This could be justified by the scarcity of breeding sites favorable to the development of this species. Moreover, contrary to *An. gambiae* s.l., *An. nili* group was found to be exophagic (significantly higher biting activity outdoors compared to indoors) in Kérou. This low biting behaviour of *An. nili* group inside the dwellings had also been reported in Nigeria [39]. Kérou being a savanna area, this behaviour of *An. nili* group confirms the results of other authors who have shown that savanna populations of *An. nili* group are more exophagic and exophilic [40, 41].

In Kérou, each inhabitant receives 5 bites of *An. nili* group per night. This biting rate was significantly higher than the one of *An. funestus* group and not negligible for just four nights of collection. This reflects the presence of breeding sites favorable to the development of this species in the area. In fact, Kérou is close to several rivers, and the water level of these rivers is kept high for a good part of the year by the rain. This flow of water contributes to the development of several permanent mosquito breeding sites. This could probably explain the high density of the populations of the *An. nili* group in Kérou. As a result, in an area with multiple vectors, variation in ecological, spatial and temporal parameters directly influences the relative role of each species in malaria transmission [42]. The biting rate of *An. gambiae* s.l. was very high in Kérou (26.19 bites/human/night) and in Péhunco (4 bites/human/night) compared to the other species and allows this vector to maintain the transmission of malaria in both districts.

Another important aspect of the current study is the involvement of *An. nili* group in malaria transmission in Benin. Indeed, the SI of *An. nili* group was 1.25% in Kérou. This index is similar to that recorded by Carnevale et al. [35] in Cameroon and lower than the one of Elissa et al. [43] in Gabon and Adja et al. [31] in Côte d’Ivoire. The sporozoite indexes of *An. gambiae* s.l. in Kérou (1.7%) and Péhunco (0.9%) were lower than those reported by previous studies in different areas of Benin [16, 33, 37]. This could be due to the low number of tested mosquitoes in the short collection period covered by the current study as compared to previous records which considered a longer time period.

This study also showed malaria transmission by different mosquito species in the Kérou district. *An. gambiae* s.l. and *An. nili* group share in the indoors and outdoors malaria transmission. In *An. funestus* group where EIR has been observed indoors, no definitive conclusion can be drawn as to its participation in outdoor transmission since the collection period in this study is short. As previously found in several sites in Benin [13–18], *An. gambiae* s.l. also remains the primary vector of malaria transmission in Kérou (13.05 infected bites/human/month), followed by *An. nili* group (1.87 infected bites/human/month) and *An. funestus* group (0.94 bites/human/month).

The EIR by *An. gambiae* s.l. in Kérou was higher than those reported in a longitudinal study by Salako et al. [37] in similar bioecological areas (Kandi and Gogounou) in the department of Alibori, Benin. In Péhunco, each inhabitant receives 1.02 infected bites of *An. gambiae* s.l. per month. The high transmission by *An. gambiae* s.l. in Kérou compared to both *An. funestus* group and *An. nili* group may be due to its significantly higher physiological age compared to the one of the other mosquito species (p < 0.05). The small number of *An. funestus* group collected in each district could explain its low level of malaria transmission. Findings of this study shows that no specimen of *An. nili* group and *An. funestus* group was collected indoors after PSC. This could be due to the low sampling of mosquitoes performed during our survey. *An. gambiae* s.l. which was the most frequent vector in the two districts takes mostly its blood meal on humans, thus confirming its anthropophagic nature [33].

Regarding the presence of resistance mechanisms in *An. gambiae* s.l., the L1014F *Kdr* mutation was found at very high frequencies in its two detected sibling species (*An. gambiae* and *An. coluzzii*) in the two districts. Contrary to the works of Gnanguenon et al. [44], Yahouédo et al. [45] and Akogbeto et al. [33] in some locations of southern and northern Benin, no significant difference was noted between the allelic frequencies of the L1014F *kdr* mutation of *An. gambiae* and *An. coluzzii* in Kérou and Péhunco. This result corroborates those of Salako et al. [46] who also found no significant difference between the L1014F *kdr* frequencies of *An. gambiae* and *An. coluzzii* in Djougou, Ségbana and Copargo, northern Benin. The trend was the same for the relatively low allelic frequencies of the G119S *ace*-*1* mutation of *An. gambiae* and *An. coluzzii* of the two study districts. However, the highest allelic frequencies of the G119S *Ace*-*1* mutation was recorded in *An. gambiae* in each zone with values ranging from 5 to 7.3%. In *An. coluzzii*, this frequency varied from 0% in Péhunco to 5% in Kérou.

## Conclusion

In the present study, *An. gambiae* s.l., *An. funestus* group and *An. nili* group were the three most important vectors that transmit malaria in the Kérou district. This is the first report on the contribution of the *An. nili* group as a secondary vector of malaria transmission in Benin. This suggests broader studies involving other districts to determine if other *Anopheles* species also contribute to malaria transmission. This will help a better orientation of vector control interventions.

## Abbreviations

HLC: human landing catches
HBR: human biting rate
PSC: pyrethrum spray catches
ELISA: enzyme-linked immunosorbent assay
PCR: polymerase chain reaction
SI: sporozoite index
EIR: entomological inoculation rate
CSP: circumsporozoite protein
